# Inhibition of the neddylation E2 enzyme UBE2M in macrophages protects against *E. coli-*induced sepsis

**DOI:** 10.1016/j.jbc.2024.108085

**Published:** 2024-12-13

**Authors:** Xuehuan Wen, Songjie Bai, Guirun Xiong, Huiqing Xiu, Jiahui Li, Jie Yang, Qing Yu, Bingyu Li, Ruomeng Hu, Lanxin Cao, Zhijian Cai, Shufang Zhang, Gensheng Zhang

**Affiliations:** 1 Department of Critical Care Medicine, Second Affiliated Hospital, Zhejiang University School of Medicine, Hangzhou, Zhejiang, China; 2 Department of Oncology, The Affiliated Cangnan Hospital of Wenzhou Medical University, Wenzhou, Zhejiang, China; 3 Department of Emergency Medicine, Tongde Hospital of Zhejiang Province, Hangzhou, China; 4 Department of Intensive Care, Zhejiang Hospital, Hangzhou, Zhejiang, China; 5 Institute of Immunology, Department of Orthopedics of the Second Affiliated Hospital, Zhejiang University School of Medicine, Hangzhou, Zhejiang, China; 6 Department of Cardiology, Second Affiliated Hospital, Zhejiang University School of Medicine, Hangzhou, Zhejiang, China; 7 Key Laboratory of Multiple Organ Failure (Zhejiang University), Ministry of Education, Hangzhou, Zhejiang, China

**Keywords:** sepsis, macrophage, UBE2M, inflammatory response, *Escherichia coli*, JAK-STAT

## Abstract

UBE2M, an essential neddylation E2 enzyme, has been implicated in the pathogenesis of various diseases, including cancers, viral infections, and obesity. However, whether UBE2M is involved in the pathogenesis of bacterial sepsis remains unclear. In an *Escherichia coli* (*E. coli*)-induced sepsis mouse model, increased UBE2M expression in macrophages in liver and lung tissues postinfection was observed. To further clarify the role of UBE2M in macrophages, mice with macrophage-specific deletion of UBE2M (*Lysm*^*+*^*Ube2m*^*f/f*^) were constructed. Compared with control mice, these mice presented decreased levels of proinflammatory cytokines, such as IL-1β, IL-6, and TNF-α; reduced sepsis-induced organ injury; and improved survival. Notably, macrophage-specific deletion of UBE2M did not impair *E. coli* clearance. *In vitro* experiments also revealed that UBE2M-deficient macrophages produced fewer proinflammatory cytokines after *E. coli* infection without hindering *E. coli* clearance. RNA-sequencing analysis revealed that UBE2M deletion in macrophages after lipopolysaccharide stimulation notably suppressed transcriptional activation within the JAK-STAT and Toll-like receptor signaling pathways, which was further confirmed by gene set enrichment analysis. Additionally, Western blotting results confirmed that UBE2M deletion inhibited the activation of the NF-κB, ERK, and JAK-STAT signaling pathways. In conclusion, our findings indicate that specific deletion of UBE2M in macrophages protects against *E. coli*-induced sepsis by downregulating the excessive inflammatory response, potentially providing a novel strategy against sepsis by targeting UBE2M.

Sepsis is a critical condition characterized by life-threatening organ dysfunction stemming from a dysregulated host response to infection (1). It leads to high rates of morbidity and mortality, which creates a substantial public health burden (2). In 2017, an estimated 48.9 million cases of sepsis occurred globally, resulting in approximately 11.0 million deaths, accounting for 19.7% of all worldwide fatalities (3). The host inflammatory response to invasive pathogen infections, guided by cellular and humoral immunity, can either completely eradicate infections or effectively contain them (4). However, an excessive inflammatory response might become life-threatening, as shown in sepsis (5). Our previous study revealed that the downregulation of Th1 and Th17 responses *via* autophagy protects against methicillin-resistant *Staphylococcus aureus* (*MRSA*)–induced sepsis without affecting bacterial clearance (6). The complex nature of the host inflammatory response prompted our investigation into strategies to both control inflammation and combat infection.

Frontline macrophages in tissues act as vital immune sentinels and are fundamental to the innate immune response. Their pivotal roles include initiating and modulating inflammatory responses, particularly in diseases such as sepsis, cancer, and atherosclerosis (7). Neddylation, a posttranslational modification, involves the covalent conjugation of the ubiquitin-like modifier NEDD8 to substrate proteins. The process primarily relies on one E1-activating enzyme (NAE), two E2-conjugating enzymes (UBE2M/UBC12 and UBE2F), and multiple E3 ligases (8). MLN4924, an inhibitor of the neddylation E1 enzyme NAE1, is widely used to investigate the role of neddylation (9). In macrophages, MLN4924 prevents IkBa degradation, increases the level of phosphorylated IkBa, and then represses the expression of TLR4-induced proinflammatory cytokines (TNF-α and IL-6) (10). Our previous study also revealed that neddylation was activated by *MRSA*-induced sepsis in mice, whereas the inhibition of neddylation in macrophages by MLN4924 promoted the development of *MRSA*-induced sepsis *via* the suppression of reactive oxygen species production (11). Nonetheless, MLN4924 is not a specific inhibitor of the neddylation pathway, as it also has neddylation-independent effects, including ciliogenesis inhibition and glycolysis promotion (12). Thus, whether and how specific neddylation pathways in macrophages are involved in the pathogenesis of sepsis remain unknown.

UBE2M, a crucial neddylation E2 enzyme downstream of NAE1, is instrumental in multiple cellular processes, such as cell cycle progression, the DNA damage response, and protein degradation (13, 14), and is involved in the pathogenesis of various diseases, including cancers and viral infections (14, 15). In intrahepatic cholangiocarcinoma, UBE2M knockdown reduces cell viability and tumor growth by inducing DNA damage responses and apoptosis (15). Our previous study revealed that UBE2M protects RIG-I from degradation by preventing its interaction with the E3 ubiquitin ligase STUB1, consequently activating antiviral IFN-I signaling. In turn, IFN-I signaling–activated STAT1 enhances Trim21 transcription, leading to an increase in UBE2M degradation and a decrease in antiviral immunity in viral infections (16). However, whether UBE2M is involved in bacterial-induced sepsis remains largely unknown.

Given the critical role of UBE2M in macrophages in the pathogenesis of viral infections and the fact that *Escherichia coli* (*E. coli*) is a predominant causative pathogen of sepsis (17), we hypothesized that UBE2M in macrophages plays an important role in the development of *E. coli*-induced sepsis. In this study, we investigated the effect of UBE2M in macrophages on *E. coli*-induced sepsis.

## Results

### *E. coli* infection promotes Ube2m expression and neddylation activation

**Figure 1:**
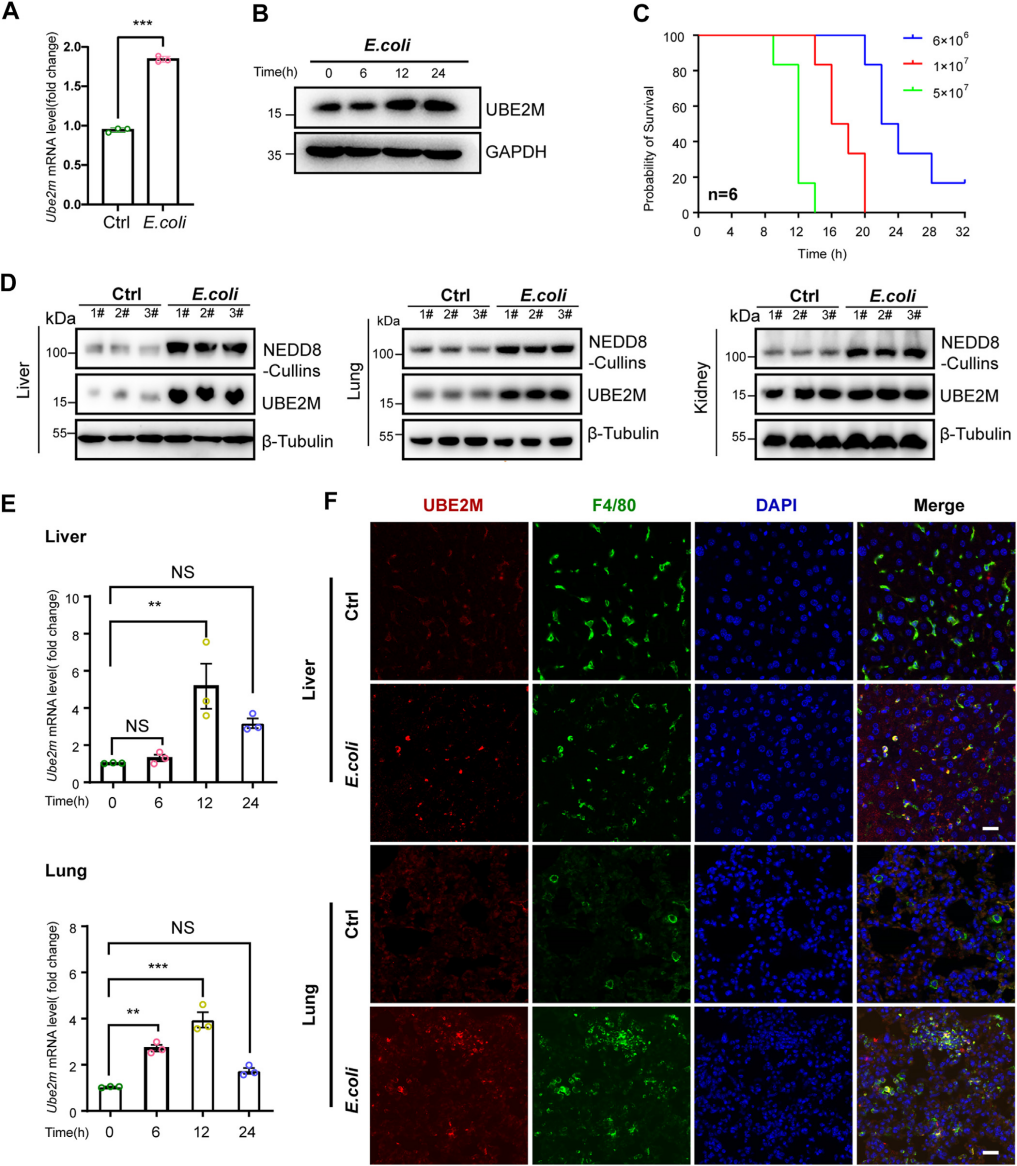
**Increased *Ube2m* expression after *E. coli* infection *in vitro and in vivo*.***A–B*, peritoneal macrophages were stimulated with *E. coli* (MOI of 10:1) for 12 h, and UBE2M expression levels were subsequently tested. *A,* RT‒qPCR quantification of *Ube2m* mRNA in PMs 12 h after *E. coli* incubation. *B,* immunoblotting assessment of the UBE2M protein at the indicated time points after *E. coli* incubation. *C,* survival analysis of mice with sepsis induced by different doses of intraperitoneal injection of *E. coli. D–F,* mice were injected intraperitoneally with 6 × 10^6^ CFUs/ml *E coli* to induce sepsis. The liver, lungs, and kidneys were harvested for further assessment. *D,* immunoblotting analysis of UBE2M and NEDD8-Cullins in harvested liver, lung, and kidney tissues 12 h postinfection. *E,* time course of *Ube2m* mRNA in liver and lung tissues at the indicated time points postinfection *via* RT‒qPCR*. F,* UBE2M expression in liver and lung macrophages was evaluated by immunofluorescence 24 h after *E. coli* incubation. Scale bar, 20 μm. The data are representative of three independent biological replicates, with individual data points shown in the graphs. NS, not significant; ∗∗*p* < 0.01 and ∗∗∗*p* < 0.001 (unpaired two-tailed Student’s *t* test; mean ± SD). CFU, colony-forming unit.

To examine the effects of *E. coli* infection on UBE2M expression in macrophages, peritoneal macrophages (PMs) were exposed to *E. coli* at a multiplicity of infection of 10:1 for 12 h. This exposure led to significant increases in the expression of *Ube2m* mRNA at 12 h postinfection and Ube2m protein levels at 12 and 24 h postinfection (Fig. 1, *A* and *B*). Analysis of neddylated E1 components revealed upregulated UBA3 expression at both the mRNA and protein levels (Fig. S1, *A* and *D*), whereas NAE1 expression remained unchanged (Fig. S1, *B* and *D*). The expression of the alternative E2 enzyme, UBE2F, did not significantly change (Fig. S1, *C* and *D*). We then determined the optimal dosage of *E. coli* for *in vivo* experiments by assessing mouse survival rates after intraperitoneal injection of different dosages of *E. coli*. Initial mortality occurred 8 h after injection, with 5 × 10^7^ colony-forming units (CFUs) of *E. coli* and 20% surviving at 12 h. However, a dosage of 6 × 10^6^ CFUs resulted in 100% survival at 12 h, which decreased to 30% by 24 h (Fig. 1*C*). Therefore, a dosage of 6 × 10^6^ CFUs of *E. coli* and a 12-h postinfection time point were selected for further *in vivo* studies. Mice with *E. coli*-induced sepsis presented a significant increase in the protein expression of UBE2M in the liver or lung but not in the kidney (Fig. 1*D*). Consistent with our previous study (11), *E. coli* infection also increased neddylation activation, as evidenced by increased protein expression of NEDD8-Cullins in the liver, lung, and kidney of mice (Fig. 1*D*).

Considering the substantial increase in UBE2M expression after *E. coli* infection, the organs of the liver and lung were selected for subsequent experiments. The expression of *Ube2m* mRNA in liver and lung tissues increased at 6 h, peaked at 12 h, and then declined by 24 h (Fig. 1*E*). Consistently, confocal microscopy revealed a significant increase in UBE2M protein expression in liver and lung tissues 24 h after *E. coli* infection (Fig. 1*F*). Notably, the increased expression of UBE2M clearly colocalized with F4/80, a well-known macrophage marker (see Fig. 1*F*). Collectively, these findings suggest that *E. coli* infection promotes *Ube2m* expression in macrophages both *in vitro* and *in vivo*.

### UBE2M deficiency in macrophages attenuates inflammation and organ injuries caused by *E. coli* infection without impairing bacterial clearance

**Figure 2:**
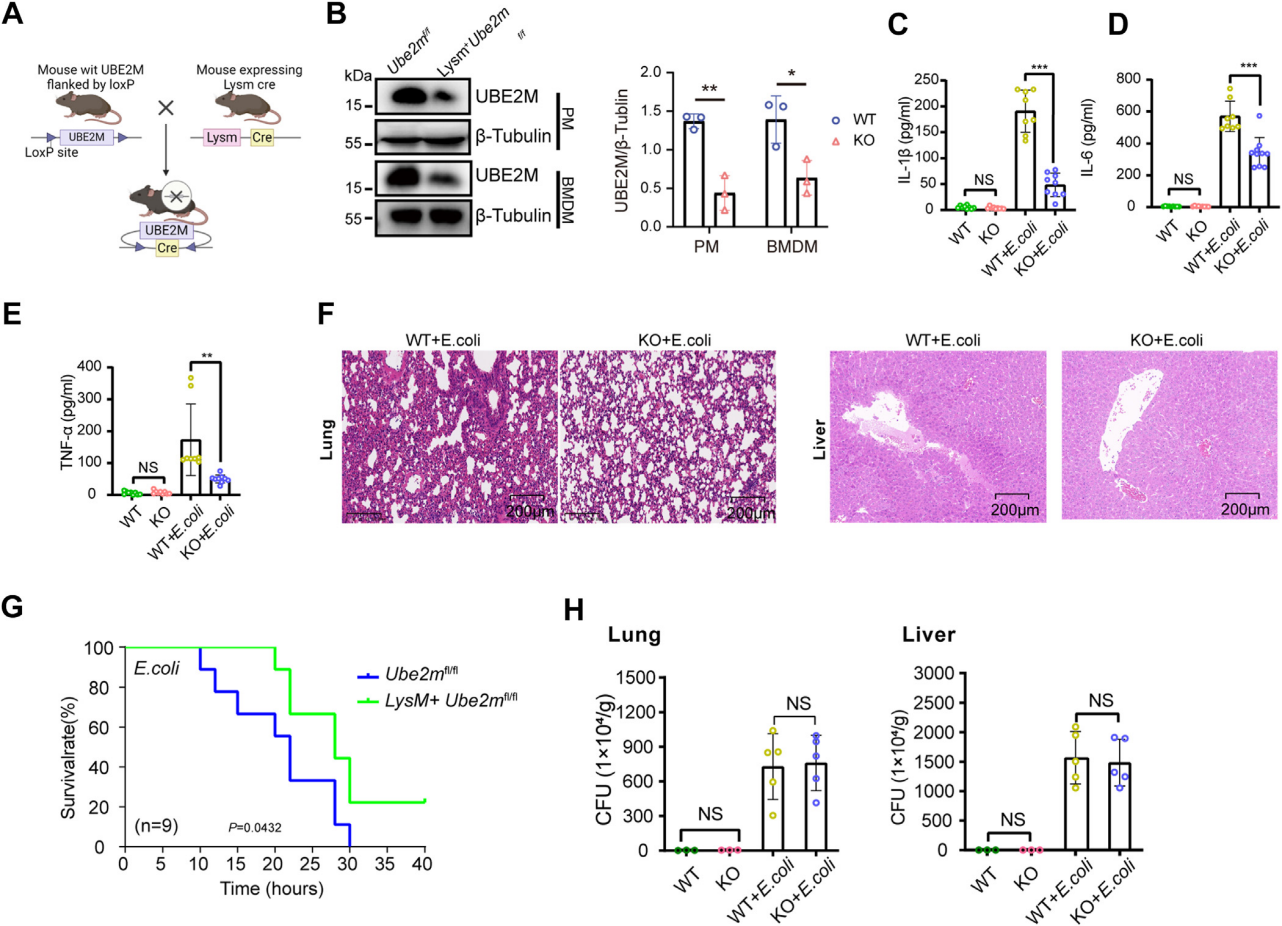
**UBE2M deficiency in macrophages has a protective effect on mice with *E. coli*-induced sepsis but has no influence on *E. coli* clearance.***A,* schematic showing the generation of macrophage-specific UBE2M knockout mice by crossing Ube2m^f/f^ with Lysm-cre mice. *B,* Western blotting of UBE2M in peritoneal macrophages (PMs) and bone marrow–derived macrophages (BMDMs) from *Ube2m*^*f/f*^ and *Lysm*^*+*^*Ube2m*^*f/f*^ mice. *C–H,* sepsis was induced in *Ube2m*^*f/f*^ and *Lysm*^*+*^*Ube2m*^*f/f*^ mice through an intraperitoneal injection of *E. coli*. The corresponding indicators were subsequently compared. *C–E,* proinflammatory cytokines (IL-1β, IL-6, and TNF-α) in the serum were measured *via* ELISA. *F,* histopathological analysis of lung and liver tissues. *G,* survival analysis of mice with or without UBE2M deficiency (n = 9 in each group). *H,* assessment of the bacterial load in lung and liver tissues. The data are representative of three independent biological replicates, with individual data points shown in the graphs. NS, not significant; ∗*p* < 0.05, ∗∗*p* < 0.01, and ∗∗∗*p* < 0.001 (unpaired two-tailed Student’s *t* test; mean ± SD).

To clarify whether macrophage UBE2M affects the progression of sepsis, we crossed Lysm-Cre (*Lysm*^*+*^) mice with *Ube2m*^*f/f*^ mice to obtain mice with UBE2M-deficient myeloid cells (*Lysm*^*+*^*Ube2m*^*f/f*^ mice) (Fig. 2*A*). The *Lysm*^*+*^*Ube2m*^*f/f*^ mice exhibited pronounced UBE2M deficiency in macrophages, as validated by immunoblot analysis (Fig. 2*B*). Compared with *Ube2m*^*f/f*^ mice, *Lysm*^*+*^*Ube2m*^*l/f*^ mice with *E. coli-*induced sepsis presented significantly lower levels of serum proinflammatory cytokines (IL-1β, IL-6, and TNF-α) (Fig. 2, *C*–*E*), markedly alleviated alveolar wall thickening, lung edema, and liver injury (Fig. 2*F*), and an obvious improvement in the overall survival rate (Fig. 2*G*). Notably, the bacterial loads in the lungs and livers of mice with *E. coli*-induced sepsis were similar regardless of macrophage-specific UBE2M deficiency (Fig. 2*H*). These findings indicate that UBE2M deficiency in macrophages results in reduced inflammation during sepsis without affecting the clearance of *E. coli,* contributing to improvements in lung injury and survival in mice with *E. coli*-induced sepsis.

### UBE2M deficiency in macrophages attenuates proinflammatory cytokine secretion

**Figure 3:**
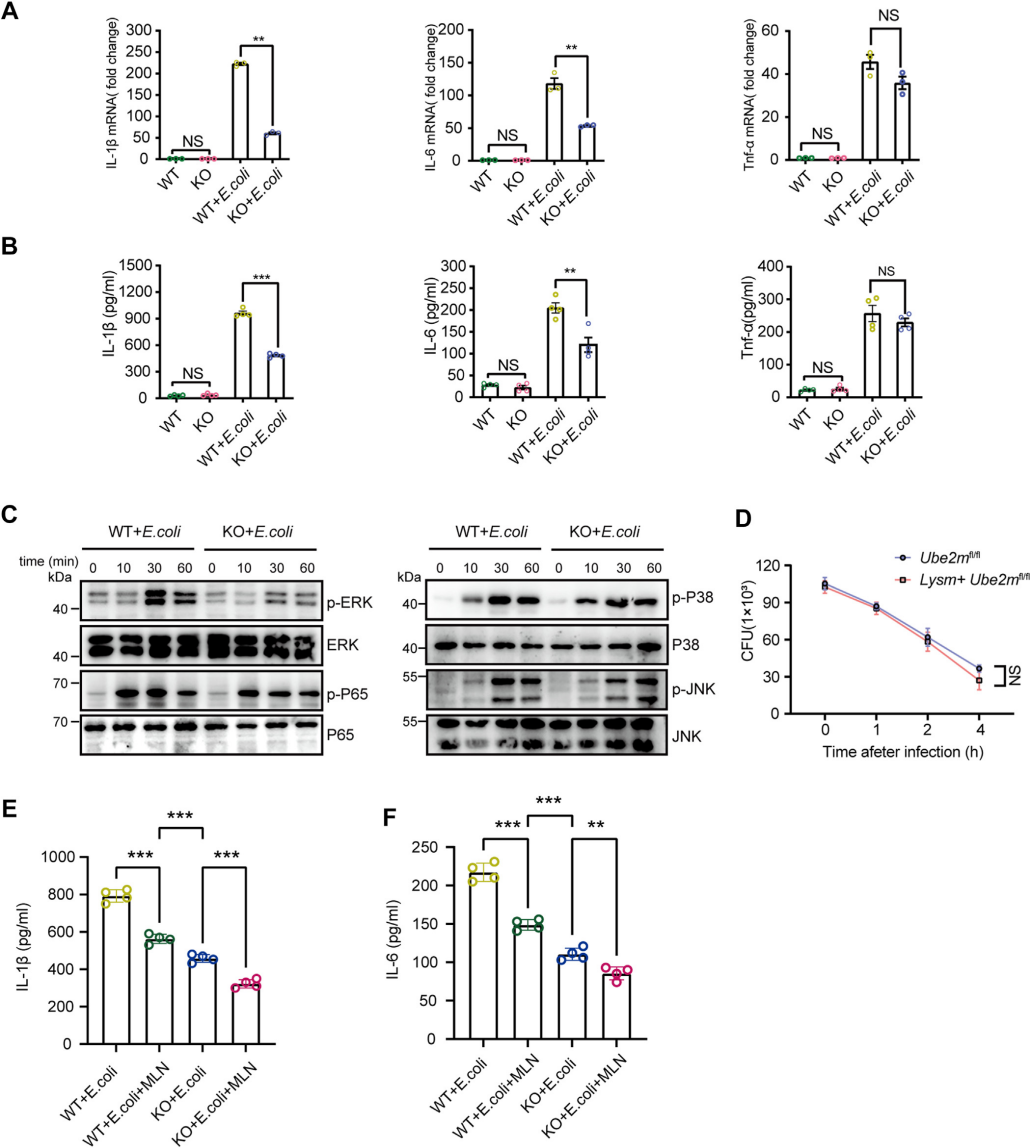
**UBE2M deficiency reduces proinflammatory cytokine production through the NF-κB and ERK pathways without affecting *E. coli* clearance in macrophages.** PMs from both groups of *Ube2m*^*f/f*^ and *Lysm+Ube2m*^*f/f*^ mice were exposed to *E. coli* for 12 h or the indicated time points before the following assessment. *A and B*, mRNA levels of the cytokines IL-1β, IL-6, and TNF-α in PMs (A) and protein levels of the cytokines IL-1β, IL-6, and TNF-α in the supernatants (B) were measured *via* RT‒qPCR or ELISA, respectively. *C,* the levels of phosphorylated ERK, P65, P38, and JNK in PMs were measured *via* Western blotting. *D,* the bactericidal capacity of PMs at the indicated times. *E and F,* PMs were treated with or without MLN4924 (100 nM) for 6 h before *E. coli* stimulation. The protein levels of IL-1β (E) and IL-6 (F) in the supernatants were measured by ELISA after 12 h of *E. coli* exposure. The data are representative of three independent biological replicates, with individual data points shown in the graphs. NS, not significant; ∗∗*p* < 0.01 and ∗∗∗*p* < 0.001 (unpaired two-tailed Student’s *t* test; mean ± SD). PM, peritoneal macrophage.

Consistent with the *in vivo* findings, *in vitro* experiments revealed that UBE2M-deficient macrophages presented significant downregulation of IL-1β and IL-6 at both mRNA level (in cells) and protein level (in culture supernatant) when stimulated with *E. coli*, whereas TNF-α expression slightly but not significantly decreased (Fig. 3, *A* and *B*). To investigate whether UBE2M in macrophages plays a critical role in the regulation of proinflammatory signaling pathways, the NF-κB and MAPK pathways were detected in both UBE2M-deficient and wildtype macrophages. Significant decreases in the phosphorylation of P65 and ERK were observed in UBE2M-deficient macrophages after *E. coli* exposure, whereas p-P38 and p-JNK expression was similar in macrophages with or without UBE2M deficiency (Fig. 3*C*). Interestingly, there was no difference in the bactericidal capacity against *E. coli* between UBE2M-deficient and wildtype macrophages (Fig. 3*D*). These results imply that UBE2M deficiency inhibits the inflammatory response to *E. coli* infection in macrophages *via* partial suppression of the NF-κB and ERK pathways but does not influence their bactericidal capacity.

To investigate whether the role of UBE2M in inflammation depends on its neddylation activity, we examined IL-1β and IL-6 production in response to *E. coli* stimulation. Wildtype macrophages exhibited robust IL-1β production following *E. coli* challenge. Compared with wildtype cells, UBE2M deletion significantly decreased IL-1β production by approximately 50%. Similarly, inhibition of neddylation with MLN4924 in wildtype cells resulted in comparable reductions in IL-1β levels. Notably, MLN4924 treatment of UBE2M-deficient macrophages further suppressed IL-1β production beyond the effect of UBE2M deletion alone (Fig. 3*E*). This pattern was also observed for IL-6 production (Fig. 3*F*), suggesting that UBE2M regulates inflammatory responses through both neddylation-dependent and neddylation-independent mechanisms.

### RNA sequencing confirmed the role of UBE2M in regulating the inflammatory response

**Figure 4:**
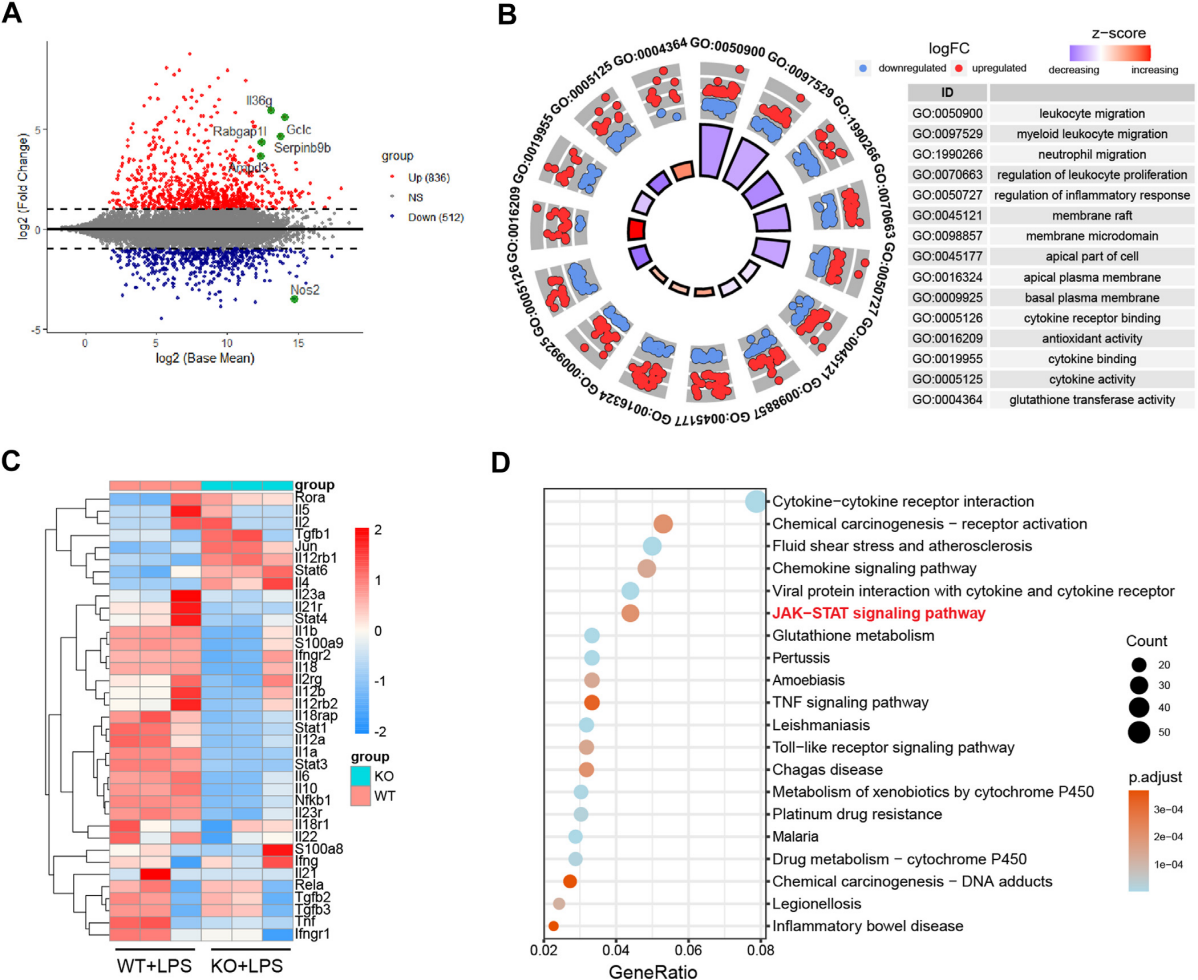
**RNA sequencing analysis of *Lysm***^***+***^***Ube2m***^***f/f***^**and *Ube2m***^***f/f***^**PMs following LPS treatment.** PMs derived from *Ube2m*^*f/f*^ and *Lysm+Ube2m*^*f/f*^ mice were treated with LPS for 6 h, after which total RNA was extracted for sequencing. *A,* scatter plot displaying the detected genes; the x-axis shows log2 base expression, and the y-axis shows the log2-fold change. Thresholds were set at |log_2_FC|>1 and adjusted *p* value < 0.05. *B,* circular plot visualizing GO analysis of differentially expressed genes (DEGs). The outer labels highlight the GO terms (left) and their corresponding annotations (right). Jitter points represent enriched genes, with upregulated genes in *red* and downregulated genes in *blue*. The internal circle depicts the z-score of each term. *C,* heatmap demonstrating the expression of selected inflammation-associated genes in the signature. *D,* KEGG enrichment results of DEGs. KEGG, Kyoto Encyclopedia of Genes and Genomes; PM, peritoneal macrophage; GO, Gene Ontology.

To further clarify the effect of UBE2M on the inflammatory response, we conducted RNA-sequencing analysis of *Ube2m*^*f/f*^ and *Lysm*^+^*Ube2m*^*f/f*^ PMs 6 h after lipopolysaccharide (LPS) stimulation. The analysis revealed 1348 differentially expressed genes (DEGs), with 836 upregulated and 512 downregulated genes in *UBE2M*-deficient macrophages (Fig. 4*A*). Gene Ontology (GO) analysis revealed that the DEGs were associated primarily with the down-regulation of inflammatory responses, leukocyte migration, and proliferation (Fig. 4*B*). Additionally, these DEGs were found to be associated with specific cellular regions, particularly those linked with the cell membrane. Enrichment within the molecular function category indicated a crucial role of UBE2M in cytokine interactions, with significant enrichment in “cytokine receptor binding”, “cytokine binding”, and “cytokine activity” (Fig. 4*B*). Moreover, the prevalence of DEGs associated with “antioxidant activity” and “glutathione transferase activity” combined with a general trend of upregulated gene expression underscores the potential importance of UBE2M in oxidative stress protection (Fig. 4*B*). Considering the inhibitory role of UBE2M in proinflammatory cytokine production, we investigated its impact on a previously reported inflammation-associated gene signature (18). The heatmap reveals a notable reduction in these genes in UBE2M-deficient macrophages compared with wildtype controls. (Fig. 4*C*). Consistent with the GO analysis results, Kyoto Encyclopedia of Genes and Genomes (KEGG) pathway enrichment revealed significant enrichment in cytokine-related pathways, such as "viral protein interaction with cytokine and cytokine receptor", "cytokine‒cytokine receptor interaction", "JAK‒STAT signaling pathway", and "TNF signaling pathway" (Fig. 4*D*). In addition, pathways related to pathogen infection and metabolism, such as "Malaria", "Leishmaniasis", "Pertussis", and "Glutathione metabolism", were also prominently enriched (Fig. 4*D*). Together, these results suggest a crucial role of UBE2M in regulating both inflammatory responses and metabolism-associated pathways.

### UBE2M modulates the JAK-STAT signaling pathway in macrophages

**Figure 5:**
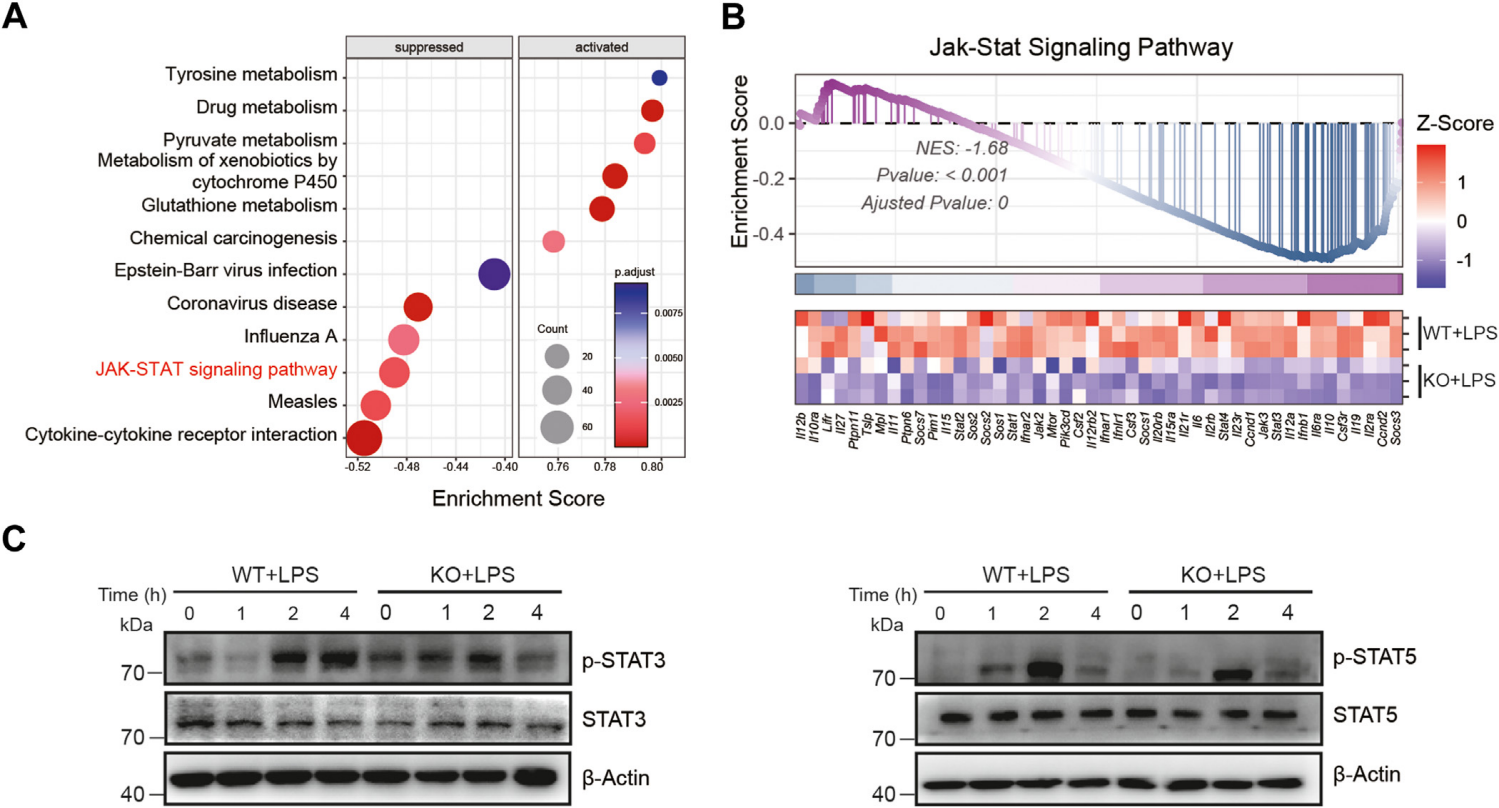
**GSEA of RNA-seq data focused on the JAK‒STAT pathway, and the results were validated by Western blotting.***A,* GSEA identified the top six suppressed and activated pathways, highlighting the role of the JAK-STAT pathway. *B*, presentation of the JAK-STAT pathway enrichment curve alongside its heatmap of the leading edge genes. *C*, Western blot analysis of the indicated proteins in *Lysm*^*+*^*Ube2m*^*f/f*^ and *Ube2m*^*f/f*^ PMs following LPS stimulation for the indicated times. PM, peritoneal macrophage; GSEA, gene set enrichment analysis.

To gain deeper insight into the altered response of UBE2M knockout in macrophages to *E. coli* infection, we performed gene set enrichment analysis (GSEA) utilizing KEGG gene sets. GSEA revealed that cytokine‒cytokine receptor interactions, measles, and the JAK‒STAT signaling pathway were most significantly suppressed (Fig. 5*A*). In contrast, metabolism-related pathways, especially those intrinsically linked to macrophage antioxidant processes such as tyrosine metabolism, glutathione metabolism, and pyruvate metabolism, exhibited substantial activation (Fig. 5*A*). Owing to the significant enrichment and pivotal role of the JAK-STAT pathway, we generated an enrichment curve, which had a normalized enrichment score of −1.68. The corresponding heatmap displayed the leading-edge genes within the JAK-STAT pathway, the majority of which indicated generalized decreases in gene expression, including key genes such as *Jak3*, *Stat3*, and *Il6* (Fig. 5*B*). To validate these observations, we detected the expression of p-STAT3 and p-STAT5 in *Ube2m*^*f/f*^ PMs and *Lysm*^+^*Ube2m*^*f/f*^ PMs following LPS stimulation. As expected, marked decreases in p-STAT3 and p-STAT5 expression were observed in *Lysm*^+^*Ube2m*^*f/f*^ PMs (Fig. 5*C*). Overall, these findings emphasize that UBE2M deficiency results in the suppression of the JAK‒STAT signaling pathway.

## Discussion

**Figure 6:**
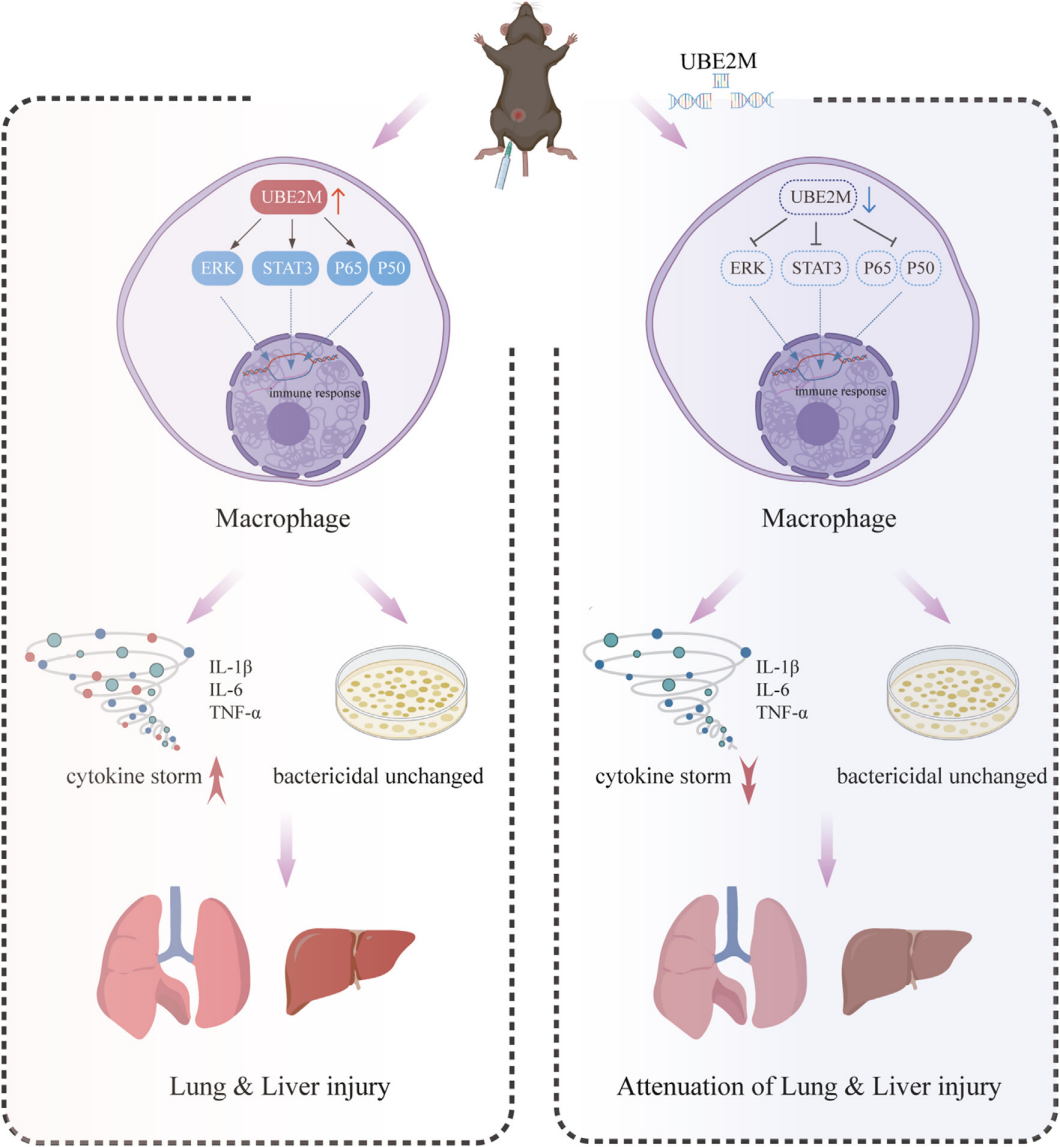
**Proposed mechanistic model illustrating the protective role of UBE2M inhibition in macrophages against lung and liver injuries in mice with *E. coli*-induced sepsis.** In response to *E. coli* infection, the expression of the UBE2M protein is upregulated in macrophages. Deficiency of UBE2M in macrophages attenuates the production of proinflammatory cytokines by downregulating the activation of the NF-κB, ERK, and JAK-STAT signaling pathways without compromising the clearance of *E. coli*.

Several novel findings were reported in the present study. First, UBE2M expression in macrophages was upregulated in mice with *E. coli*-induced sepsis. Second, macrophage-specific UBE2M deficiency attenuated *E. coli* infection–induced inflammation and organ injury in mice, whereas it did not affect *E. coli* clearance. Third, UBE2M deficiency led to reduced production of proinflammatory cytokines and downregulated the activation of the NF-κB, ERK, and JAK-STAT signaling pathways. These findings suggest that UBE2M in macrophages might play a crucial role in the pathogenesis of *E. coli*-induced sepsis (Fig. 6).

UBE2M is involved in the pathogenesis of several diseases, including hepatocellular carcinoma, osteoarthritis, viral infections, and inflammatory diseases, and UBE2M in macrophages plays a crucial role in infection and inflammation (16, 19, 20, 21). Our previous study demonstrated that vesicular stomatitis virus or influenza A (H1N1) virus infection promotes the synthesis of inflammatory cytokines, such as type I interferon, IL-6, and TNF, in macrophages and that UBE2M deficiency in macrophages significantly decreases these virus infection–induced inflammatory responses (16). In obesity, a noninfectious inflammatory disease, UBE2M contributes to elevated secretion of the inflammatory cytokines IL-1β, IL-6, and TNF, while its absence leads to reduced levels of these cytokines (22). In our current study, we found that *E. coli* infection markedly promoted the upregulation of UBE2M in macrophages. Compared with WT mice, mice with specific deficiency of UBE2M in macrophages (*Lysm*^+^*Ube2m*^*f/f*^ mice) presented significant decreases in inflammatory responses and improvements in the survival rate after *E. coli*-induced sepsis. Taken together, these results suggest that the inhibition of UBE2M in macrophages is critically involved in protection against inflammatory diseases, including infectious or noninfectious diseases, and our study further expands the biological significance of UBE2M in bacterial sepsis, as no such study on the role of UBE2M in macrophages in bacterial infections has been reported.

The NF-κB and ERK pathways are both vital in the control of inflammatory responses (23, 24). According to the results of the RNA sequencing enrichment in the present study, UBE2M-deficient macrophages presented reduced cytokine production and an inflammatory response to LPS stimulation. Moreover, we detected a significant reduction in inflammation-associated gene signatures (18), which demonstrated that UBE2M has the capacity to regulate inflammation-related gene expression patterns. Notably, further GSEA indicated that UBE2M deficiency led to primary suppression of the JAK-STAT pathway, which was evidenced by significant decreases in the expression of p-STAT3 and p-STAT5 after LPS stimulation in UBE2M-deficient macrophages. The JAK-STAT pathway plays an important role in many inflammatory and infectious diseases (25). These findings suggest that UBE2M may play a pivotal role in modulating the NF-κB, ERK, and JAK-STAT pathways, potentially impacting the pathogenesis of various inflammatory and infectious diseases.

Macrophages serve as a pivotal first line of defense against invading pathogens, and their ability to clear these pathogens may influence the progression of sepsis (26). Another important phenomenon in the current study was that UBE2M deficiency in macrophages had no influence on their ability to clear *E. coli*. This phenomenon can be explained by three key factors. First, while moderate inflammation facilitates pathogen clearance, excessive inflammation damages host tissues and impairs pathogen elimination. UBE2M deletion suppressed excessive inflammation while maintaining beneficial inflammatory responses, thus preserving bacterial clearance capacity (27). Second, inflammatory responses and bacterial clearance in macrophages operate through partially independent molecular pathways—UBE2M primarily modulates inflammatory signaling (*e.g.*, the NF-κB pathway) without significantly affecting phagocytic and bactericidal mechanisms. Third, *in vivo* bacterial burden regulation involves complex interactions among immune and nonimmune cells. The overall outcome is a result of this complex interplay. In addition, previous studies have indicated that UBE2M deficiency in macrophages increases the susceptibility of mice to herpes simplex virus 1 (16), vesicular stomatitis virus, and H1N1 infection (16, 28). These results suggest that the effect of UBE2M in macrophages on the clearance of pathogens by macrophages might be dependent on different pathogen species. However, more evidence is needed to confirm the different roles of UBE2M in macrophages in the pathogenesis of various infections caused by different pathogens, and the exact mechanisms involved also need to be further investigated.

Some limitations of our study also exist. First, the role of UBE2M in macrophages in sepsis induced by pathogens other than *E. coli* remains unknown and needs further investigation. Second, although our transition from *E. coli* to LPS stimulation was based on our initial finding of TLR4 pathway involvement and allowed for more standardized mechanistic studies, this focused approach using purified LPS may not fully represent the complex host–pathogen interactions that occur during bacterial sepsis. Third, our study focused on the role of macrophages in *E. coli*-induced sepsis, and the involvement of UBE2M in other immune cells remains unknown. Finally, we also refrained from a deeper exploration of the molecular mechanisms involved, and we will investigate these molecular interactions in detail in the future.

In conclusion, UBE2M-deficient macrophages substantially reduce inflammation and increase survival in *E. coli*-induced septic mice without compromising bacterial clearance. Mechanistically, UBE2M deficiency suppresses the activation of the NF-κB, ERK, and JAK-STAT signaling pathways. These results suggest that the inhibition of UBE2M in macrophages might be a potential therapeutic target in sepsis.

## Experimental procedures

### Bacterial preparation

The *E. coli* strain ATCC25922, sourced from Miaoling Bio (T0109), was cultured in Tryptic Soy Broth medium and incubated at 37 °C for 8 h. After cultivation, it was serially diluted with sterile phosphate-buffered saline (PBS) and plated on Tryptic Soy Agar. The number of CFUs was counted after overnight incubation at 37 °C. The bacteria were then centrifuged to produce a pellet, which was subsequently washed and resuspended in PBS for additional experiments.

### Animal subjects

Eight-week-old male C57BL/6 mice were housed under specific pathogen-free conditions at the Experimental Animal Center of Zhejiang University. We also used *Ube2m*^*flox/flox*^ mice, which were kindly provided by Professor Sun Yi's team at the Institute of Translational Medicine, Zhejiang University. The studies were approved by the Ethics Committee for Animal Studies at Zhejiang University.

### Establishment of a murine sepsis model via intraperitoneal injection of *E. coli*

A mouse model of *E. coli-induced* sepsis was established *via* intraperitoneal inoculation of the live ATCC25922 strain, as detailed in a previous study (29).

### Isolation and purification of mouse PMs

PMs were isolated from 8-week-old C57BL/6 mice 3 days after intraperitoneal injection with 2 ml of 3% sterile thioglycolate medium, as previously described (11). Each mouse was euthanized using pentobarbital sodium, and the abdomen was sterilized with 70% ethanol. The peritoneal cavity was flushed with ice-cold RPMI 1640 medium through a small incision. The extracted peritoneal fluid was centrifuged at 250×*g* for 5 min at 4 °C, and the resulting cell pellet was resuspended in RPMI 1640 medium enriched with 10% FBS and 100 U/ml penicillin. The cell count was then determined. The cells were subsequently plated at a density of 5 × 10^5^ cells/ml on either 12- or 24-well tissue culture plates and incubated for 2 h at 37 °C, after which nonadherent cells were gently rinsed off with PBS, and purified adherent macrophages were obtained.

### Histopathological analysis

The liver and lung tissues of the mice were harvested 12 h after *E. coli* injection, promptly fixed in 4% paraformaldehyde, and stained with hematoxylin and eosin. For each mouse, a minimum of three randomly selected microscopic fields from each slide were captured.

### Cytokine analysis

The concentrations of the cytokines IL-6 (Cat No. 431304), IL-1β (Cat No. 432601), and TNF-α (Cat No. 430907) were assessed *via* ELISA kits from BioLegend. The procedures were performed according to the manufacturer's instructions.

### Immunofluorescence staining and confocal microscopy

Liver and lung tissues were rapidly frozen in liquid nitrogen for 30 min and then stored at −80 °C. The tissues were sectioned on ice, fixed in precooled 4% paraformaldehyde for 20 min, permeabilized with 0.1% Triton X-100 for 10 min, rinsed thrice with PBS with Tween 20 (PBST), and blocked with goat serum for 1 h. The sections were incubated overnight at 4 °C with primary antibodies against UBE2M (Abcam, ab109507) and F4/80 (Tonbo Biosciences, 35–4801-U100). After being washed with PBS, the sections were incubated for 1 h with goat anti-rabbit IgG DyLight594 (red; MultiSciences, 70-GAR5942) for UBE2M detection and with goat anti-mouse IgG DyLight488 (green; MultiSciences, 70-GAM4882) for F4/80 detection. Nuclei were counterstained with DAPI (blue). The stained sections were observed under a Nikon confocal microscope, and the images were analyzed with ImageJ.

### Immunoblotting procedure

Cell lysate proteins (20 μg) were separated on 10% SDS‒PAGE gels and electrotransferred onto polyvinylidene difluoride membranes. The membranes were blocked with 5% nonfat milk in PBS with Tween 20 and incubated overnight at 4 °C with the following primary antibodies: anti-UBE2M (Abcam, ab109507), anti-NEDD8 (Abcam, ab81264), anti-β-tubulin (ABclonal, AC021), anti-GAPDH (Abways, AB0037), anti-UBE2F (Abcam, ab87640), anti-phospho-ERK (Bioworld, bs5016), anti-ERK (Proteintech, 51068-1-AP), anti-phospho-P65 (CST, 3033), anti-P65 (CST, 8242s), anti-phospho-P38 (CST, 9216s), anti-P38 (ABclonal, A14401), anti-phospho-JNK (CST, 4671 s), and anti-JNK (CST, 9252). After washing, the bound antibodies were detected with HRP-conjugated secondary antibodies (goat anti-mouse IgG HRP, MultiSciences, 70-GAM007 or goat anti-rabbit IgG HRP, MultiSciences, 70-GAR007; 1:5000) for 1 h, and the signal was visualized using enhanced chemiluminescence reagents. Visualization and imaging were executed with a Tanon 4500 gel imaging system. For quantitative analysis, the band intensities were measured using ImageJ software (NIH). The relative protein levels were normalized to their respective loading controls (β-tubulin). Statistical analysis was performed on data from three independent experiments using unpaired Student's *t* test. *p* < 0.05 was considered statistically significant.

### Quantitative real-time PCR

Total RNA was extracted using TRIzol reagent (15596026, Thermo Fisher Scientific) according to the manufacturer's guidelines, and the isolated RNA was reverse-transcribed into cDNA using a cDNA synthesis kit (CW2569, Cowin Biotech). β-actin was used as a housekeeping gene for normalization. Real-time PCR was performed *via* SYBR Green (CW0955, Cowin Biotech) on an Applied Biosystems 7500 real-time PCR system (Thermo Fisher Scientific). The sequences of the primers used are listed in Table 1.

**Table 1:** Primer sequences used in this study

Target	Direction	Sequence (5′ → 3′)
*TNF-α*	Forward	CAAACCACCAAGTGGAGGAG
*TNF-α*	Reverse	GTGGGTGAGGAGCACGTAGT
*IL-6*	Forward	CTGCAAGAGACTTCCATCCAG
*IL-6*	Reverse	AGTGGTATAGACAGGTCTGTTGG
*IL-1β*	Forward	GCAACTGTTCCTGAACTCAACT
*IL-1β*	Reverse	ATCTTTTGGGGTCCGTCAACT
*Uba3*	Forward	GGAGACTGGGAAGGTCGCT
*Uba3*	Reverse	AGAACTGGAGTGATTCAGTGCT
*Ube2f*	Forward	ACGCTGGCAAGCAAGTTGA
*Ube2f*	Reverse	CCTCATCTGGGCTTACAGTCAG
*Ube2m*	Forward	AACCTGCCCAAGACGTGTG
*Ube2m*	Reverse	AGCTGAATACAAACTTGCCACT
*Nae1*	Forward	ACTCAAGGAGCAAAGTACGAC
*Nae1*	Reverse	TTCCTGTAGCCGTTGCATTTAT

### Measurement of bacterial load in tissues

The *E. coli-*induced septic mice were euthanized 12 h postinduction. Liver and lung tissues were subsequently collected for bacterial load analysis, as described previously (30). These tissues were placed in sterile EP tubes containing 1 ml of sterile PBS and thoroughly homogenized to create a uniform tissue suspension. From this suspension, 100 μl aliquots were taken and serially diluted 1:10 in PBS until a 10^-6^ dilution was achieved. Each dilution was spread carefully onto predried Luria‒Bertani agar plates using a sterile glass spreader, and the plates were incubated at 37 °C overnight. On the following day, the number of CFUs per dilution was counted, and the bacterial load was calculated as CFUs per gram of tissue *via* the following formula: CFUs/weight (g) = (CFU count from 0.1 ml × dilution factor × 10)/tissue weight (g).

### Assessment of macrophage bacterial killing efficacy

The macrophages were seeded at a density of 2 × 10^5^ cells per well in 24-well plates. *E. coli* was then introduced at a multiplicity of infection of 10:1 and allowed to interact with the macrophages for a 30-min stimulation period. Next, the supernatant was carefully discarded, the cells were washed three times with sterile PBS, and then fresh culture medium was added to each well. At specified time points (0, 1, 2, and 4 h after incubation), the macrophages were lysed with 1 ml of sterile water, and the resulting solution was used for CFU enumeration on Luria‒Bertani agar plates. The plates were incubated at 37 °C overnight, and the CFUs were counted on the following day.

### RNA sequencing analysis

PMs from both *Ube2m*^*f/f*^ and *Lysm*^+^*Ube2m*^*f/f*^ mice were stimulated with LPS for 6 h. Total RNA was then extracted using TRIzol reagent. The RNA samples were sequenced *via* the NovaSeq 6000 high-throughput sequencing platform (Illumina) provided by KaiTai Biotech. The raw data were preprocessed by filtering out low-quality reads and removing the adaptor sequences. The cleaned reads were then aligned to the mm10 reference genome *via* HISAT2, and the transcripts for each cell were constructed independently using StringTie. DEGs were identified using the DEGseq package (31), with a significance threshold set at an adjusted *p* value < 0.05 and a |log2(fold change)| > 1.

### Enrichment analysis

To interpret the functional relevance of the identified DEGs, we embarked on an encompassing enrichment analysis deploying the clusterProfiler package (32) in R. This analysis encompassed GO, KEGG, and GSEA. The generated results were subsequently visualized *via* the GOplot and GseaVis packages (33; https://github.com/junjunlab/GseaVis).

### Statistical analyses

GraphPad Prism 8.0.2 software and R (version 4.2.2) were used for the statistical analyses. Unpaired Student’s t tests were used for comparisons between two groups. Kaplan‒Meier curves of overall survival were compared using the log-rank test. A *p* value < 0.05 indicated a statistically significant difference.

## Data availability

The RNA-sequencing data from this study are available in the Zenodo repository (https://doi.org/10.5281/zenodo.14063261).

## Supporting information

This article contains supporting information.

## Conflict of interest

The authors declare that they have no conflicts of interest with the contents of this article.
