# Supplementary material for: Inhibition of the neddylation E2 enzyme UBE2M in macrophages protects against E. coli-induced sepsis
Source: J Biol Chem. 2024 Dec 13;301(1):108085. doi: 10.1016/j.jbc.2024.108085 (PMC11780929; doi:10.1016/j.jbc.2024.108085)
Supplement: Supplementary Figure 1 [file mmc1.docx]

**
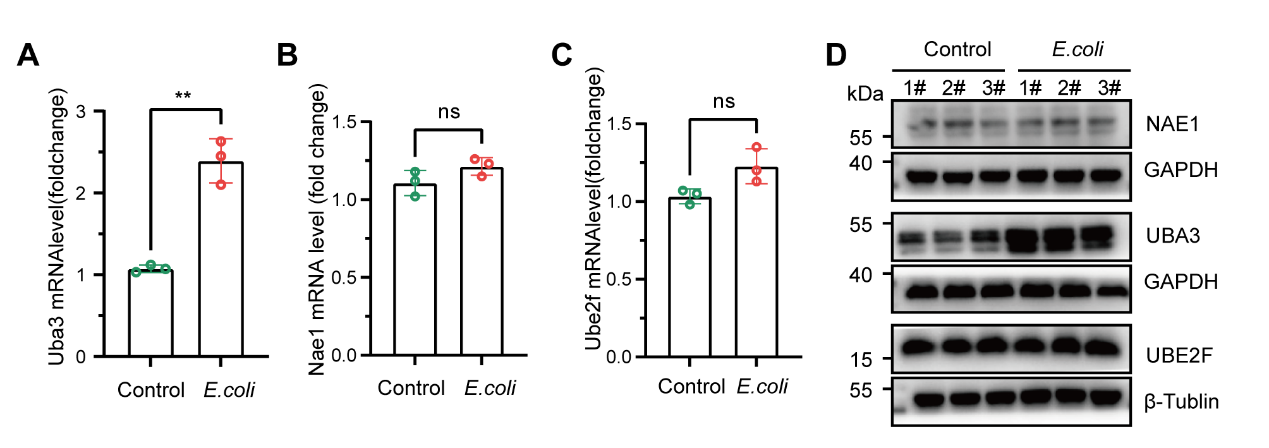
**

**Supplementary Figure 1. Expression profiling of neddylation-related enzymes in PMs upon *E. coli* infection.**

(A) *Uba3* mRNA expression in control and *E. coli*-infected peritoneal macrophages. (B) *Nae1* mRNA expression in control and *E. coli*-infected peritoneal macrophages. (C) *Ube2f* mRNA expression in control and *E. coli*-infected peritoneal macrophages. (D) Representative immunoblots showing the protein levels of NAE1, UBA3, and UBE2F in control and *E. coli*-infected PMs. The data are presented as the means ± SDs. **p < 0.01; ns, not significant.
